# Intrinsic and Extrinsic Effects of Microstructure on Properties in Cast Al Alloys

**DOI:** 10.3390/ma13092019

**Published:** 2020-04-25

**Authors:** Murat Tiryakioğlu

**Affiliations:** Jacksonville University, Jacksonville, FL 32211, USA; mtiryak@ju.edu

**Keywords:** dendrite arm spacing, pores, hot tear, β–Al_5_FeSi platelets, Si particle fracture, debonding

## Abstract

The metallurgy of cast aluminum alloys has always been considered to be different from that of wrought alloys. Metallurgists have been taught that pores are intrinsic in cast aluminum alloys and that mechanical properties in cast aluminum alloys are controlled by dendrite arm spacing, the presence of Fe-bearing particles, and the size of Si particles in Al–Si alloys, which fracture and debond during deformation, leading to premature failure. Whether these effects are intrinsic or extrinsic, i.e., mere correlations due to the structural quality of castings, is discussed in detail. Ideal properties are discussed, based on findings presented mostly in physics literature. Pores and hot tears in aluminum castings are extrinsic. Moreover, the effect of dendrite arm spacing on elongation, precipitation, and subsequent fracture of β–Al_5_FeSi platelets, and finally Si particle fracture and debonding are all extrinsic. A fundamental change in how we approach the metallurgy of cast aluminum alloys is necessary.

## 1. Introduction

Many practicing engineers have been taught to think differently about aluminum components, based on the type of final product—cast or wrought. This is due to the perceived differences in the behavior and performance of cast aluminum alloys from their wrought siblings. Although the metal is the same, cast alloys are considered to be usually inferior to wrought alloys. For instance, cast alloy A201 and wrought alloy 2014 have similar Cu and Mg contents, although Ag is also added to A201. A201 castings and 2014 forgings have similar yield and ultimate tensile strengths listed in Military Handbook 5 [1]. However, the minimum ductility for 2014 forgings is listed between 6% and 8%, whereas it is listed as 1.5% to 3% for A201 castings.

The current thinking in cast aluminum metallurgy can be summarized as follows:

- Pores are inevitable, i.e., intrinsic, in aluminum castings;
- Al–Cu alloys are difficult to cast because of their propensity for hot tears;
- The most important microstructural parameter is dendrite arm spacing, especially to control ductility;
- β–Al_5_FeSi platelets are weak and brittle, causing premature fracture in castings;
- In Al–Si alloys, Si eutectic particle size needs to be controlled (e.g., addition of modifiers such as Sr) because these particles fracture and debond in the early stages of plastic deformation, causing cracks to form, leading to premature fracture.

To the author’s knowledge, these points apply only to cast aluminum alloys, with the possible exception of the β–Al_5_FeSi platelets. This thinking has led many researchers to develop models to predict porosity and/or hot tears during solidification or to estimate mechanical properties from cast microstructure. These models have been built by ignoring the physics at least partially and making unrealistic assumptions to match the results with the observed behavior. Unfortunately, this phenomenological approach to model development continues to populate the literature with many papers that estimate the behavior of cast aluminum alloys only in some limited cases. Moreover, the observed correlations between cast microstructure and property have not been analyzed in depth. This paper is motivated by the perceived duality in aluminum metallurgy, and the need to reevaluate this thinking. An earlier version of this paper [2] has been expanded significantly based on the concepts of intrinsic and extrinsic effects in aluminum casting with additional support based on the Weibull analysis of fracture.

## 2. Intrinsic and Extrinsic Effects

The terms intrinsic and extrinsic effects in aluminum metallurgy were first introduced by J.T. Staley [3] in his “toughness tree”. Staley characterized the effects of microstructural features such as dispersoids, grain boundaries, etc., as intrinsic, and those from metallic and non-metallic inclusions that can be potentially eliminated as extrinsic. The same approach is taken in this paper. Let us now address the current thinking in the metallurgy of cast aluminum alloys.

### 2.1. Pores and Hot Tears

Pores and hot tears are two defects that appear differently and at different locations. However, the origin for both defects is the same; a pore must first nucleate under tensile stress, that is, uniaxial for hot tears and hydrostatic for pores. This essential first step for hot tear initiation was confirmed [4] via in situ observations in a transparent liquid.

**Figure 1:**
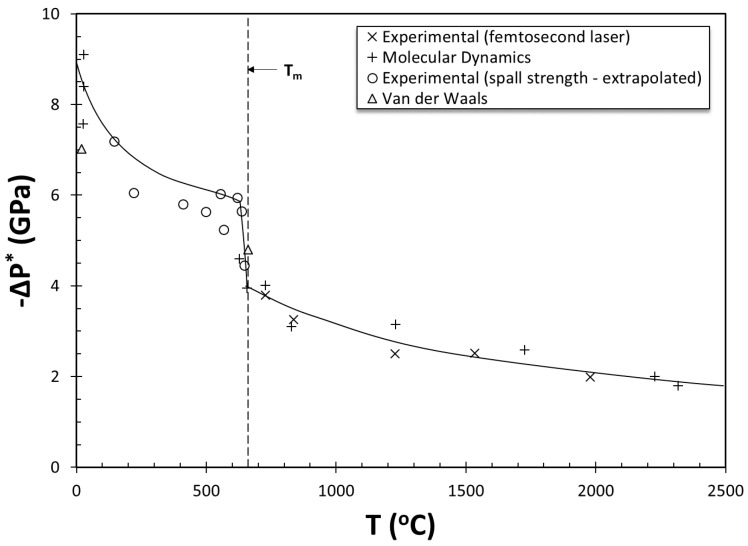
The change in fracture pressure (intrinsic strength) of liquid and solid aluminum with temperature reinterpreted based on the data from [8].

The critical radius above which a pore is stable, r^*^, for either homogeneous or heterogeneous nucleation in a liquid, i.e., is found using Equation (1):

(1) $$r^{*}=\frac{-2\sigma }{∆P^{*}}$$

where σ is the surface tension of the liquid (N.m) and ΔP^*^ is the pressure differential and is a negative number. Surface tension for liquid metals is known; for liquid aluminum at its melting temperature, σ = 1.03 N.m [5]. However, either ΔP^*^ or r* in Equation (1) needs to be estimated. In a recent review, Yousefian and Tiryakioğlu [6] showed that ΔP^*^ has been generally assumed to be −1 atm. (~ −0.1 MPa) in the literature. The intrinsic strength of liquid aluminum at its melting temperature is approximately −4 GPa. This value has been estimated [7,8] from a combination of experimental and molecular dynamics data, as presented in Figure 1. Similar results have been reported by Campbell [9,10], Shahani and Fredriksson [11], and recently by Yousefian and Tiryakioğlu. It has also been shown by the author [12] that ΔP^*^ for heterogeneous nucleation is approximately −1 GPa. These values are at least three orders of magnitude different from the assumed ΔP^*^ values in the literature. Therefore, liquid aluminum is intrinsically strong and completely resistant to pore nucleation either homogeneously or heterogeneously, even with high levels of hydrogen [6]. Hence, pore formation is an extrinsic event, and consequently pores and hot tears in aluminum castings are extrinsic.

The root cause of weakening in liquid aluminum is inclusions, specifically bifilms entrained into the bulk liquid when surface oxide films are folded over during liquid metal processing and pouring [13,14,15]. Due to these bifilms that are extrinsic, nucleation is completely bypassed, and pores form simply by the opening of bifilms [6]. There is growing evidence in the literature on the role of bifilms on causing pores [6,13,16,17,18,19,20,21,22,23,24,25,26] and leading to hot tears [27,28]. Oxides are inevitable on the surface of liquid aluminum and serve as a protective film over the liquid. However, their entrainment into the bulk liquid as bifilms can indeed be avoided by the redesign of entire casting production systems [29], mainly by eliminating pouring and any disturbance of the liquid metal surface. In clean melts, aluminum can hold hydrogen levels exceeding 300 times its solubility in solid aluminum, without forming porosity [30].

A common argument presented in the literature for hot tear formation is that grain boundaries are weak and, therefore, the unzipping of grain boundaries in the final stages of solidification, i.e., a hot tear, can be expected [31]. The solution usually suggested to eliminate hot tear in aluminum alloys is grain refining [32,33], such as adding TiB_2_ particles along with excess titanium to the melt so that aluminum can nucleate heterogeneously on these particles [34,35,36,37,38,39]. Combined with the grain growth restriction provided by Ti [40], smaller grain sizes can be achieved after grain refining. It has been argued [41] that grain refining enhances feeding characteristics of aluminum alloys during solidification. Because grain refinement results in the formation of smaller equiaxed grains, dendrite coherency, i.e., the solid fraction at which equiaxed dendrites are in contact with each other and can no longer move, is shifted to higher solid fractions during solidification. Consequently, the equiaxed grains float toward negative pressure until later stages of solidification, delaying the start of the subsequent interdendritic feeding phase. It has been assumed that the relatively shorter interdendritic channel lengths that would result from smaller grains enhances liquid metal flow through the dendritic structure further and provides feed metal where it is needed most, at the point of highest negative pressure or tensile stress. Hence, the reduction in hot tear tendency with grain refinement has been attributed to this enhanced feeding. Grain refinement, however, increases the amount of grain boundary area per unit volume. If the grain boundaries are weak, why would an increase in grain boundary area per unit volume as a result of grain refinement solve hot tear problems? We will discuss solidification characteristics, namely feeding distances, first and then address the strength of grain boundaries.

The feeding distance concept was first introduced by Pellini and co-workers [42,43,44,45] as the distance that liquid metal can travel from the feeder (alternatively known as the riser) to the point of the largest pressure drop, resulting in zero porosity in the region. Based on Darcy’s law, the author introduced a feeding distance equation [46]. Calculations of feeding distances in liquid aluminum [46] and in tin bronze [47] have shown that the metal is intrinsically capable of feeding itself during solidification through long distances (in the order of kilometers) regardless of the dendritic structure of the solid metal. Only in the presence of inclusions, i.e., entrainment defects, which weaken the liquid metal, are feeding distances greatly reduced and become sensitive to the dendritic structure. Although liquid metal is intrinsically strong, its resistance to crack propagation can be expected to be very low. Hence, the extrinsic entrainment defects give rise to pore formation, which subsequently result in cracks that form and propagate under tensile stress during solidification. 

If the liquid between grains is not extrinsically weakened by bifilms, we can consequently expect the grain boundaries to be strong upon solidification. There are only scarce experimental data on the strength of grain boundaries. In one of these rare experiments, Mikhailovskij et al. [48] mechanically annealed defect-free tungsten nanospecimens to remove all dislocations. Mechanical testing results showed that tungsten bi-crystals had a fracture strength of 20.2 GPa, which was 72% of the inherent strength of monocrystal tungsten [49]. It should be noted that the fracture strength of 20.2 GPa is more than 6 times larger than the highest fracture strength of 3 GPa, reported previously by Fridman [50] for tungsten polycrystals. Similar results have also been reported for graphene. Rasool et al. [51] found that bi-crystals with high angle grain boundaries had 89%−92% of the strength of graphene single crystals. Lee et al. [52] reported that polycrystalline graphene had 83% of the ideal strength of graphene monocrystals. These experimental findings clearly demonstrate that “clean” grain boundaries are intrinsically quite strong. 

To the author’s knowledge, there are no experimental results reported for the inherent strength of grain boundaries in aluminum. Recently, the strength of the grain boundaries in aluminum has been investigated via molecular dynamics simulations [53,54,55]. Lu et al. [53] found the inherent strength of Al monocrystals to be between 11.0 and 12.4 GPa. In the same study, the strength of a specimen with a single grain boundary was found to be 9.5 GPa, i.e., between 77% and 84% of the inherent strength of single crystal aluminum. This result is consistent with the experimental results of Mikhailovskij et al. for W, and Rasool et al. and Lee et al. for graphene. Lu et al. [55,56] also investigated the effect of Na and Ca segregation to grain boundaries. Na and Ca were found to reduce the grain boundary strength by 50% and 13%, respectively. Similar results were reported by Zheng et al. [54] for the effect of Na on grain boundary strength. Nevertheless, the grain boundary strength was still found to be at least 4 GPa, well over the bulk strength of polycrystalline aluminum. Again, we can conclude that grain boundaries, which form at the end of solidification, are intrinsically strong unless they are weakened by entrainment defects. Hence, pores and hot tears are extrinsic defects that occur due to entrainment defects.

### 2.2. Dendrite Arm Spacing–Ductility Relationship

Ductility in cast aluminum alloys has been considered to be controlled by primary (λ_1_) and especially secondary dendrite arm spacing, λ_2_ [57]. We will now review this correlation and address whether it is intrinsic or extrinsic by analyzing datasets from the literature.

The dendrite arm spacing–elongation (e_F_) relationship follows a Hall–Petch-type Equation (2) [58,59,60,61], such that:

(2) $$e_{F}={\;e}_{0}+\frac{\Lambda }{\sqrt{\lambda_{2}}}$$

A similar correlation between λ_2_ and tensile strength has been reported for various cast aluminum alloys. To normalize elongation for different levels of yield strength, we use quality index, Q_T_, calculated with the following Equation (3) [62,63,64,65,66]:

(3) $$Q_{T}=\frac{e_{F}}{\beta_{0}-\beta_{1}\cdot \sigma_{Y}}$$

Combining Equations (2) and (3), we obtain:

(4) $$Q_{T}={\;Q}_{T0}+\frac{\Lambda_{0}}{\sqrt{\lambda_{2}}}$$

**Figure 2:**
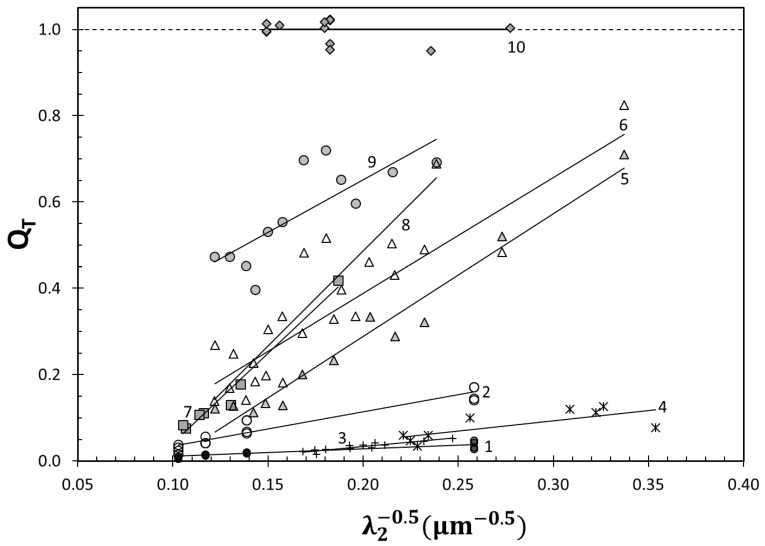
Data from ten independent studies plotted with the inverse of square root of secondary dendrite arm spacing versus the quality index, Q_T_.

Data from ten independent studies on various cast Al–Si alloys were collected. The details of the datasets analyzed in this study are presented in Table 1. The correlations between secondary dendrite arm spacing and Q_T_ for the ten datasets are presented in Figure 2. Note that the slope and intercept values are all different, which are also listed in Table 1.

**Figure 3:**
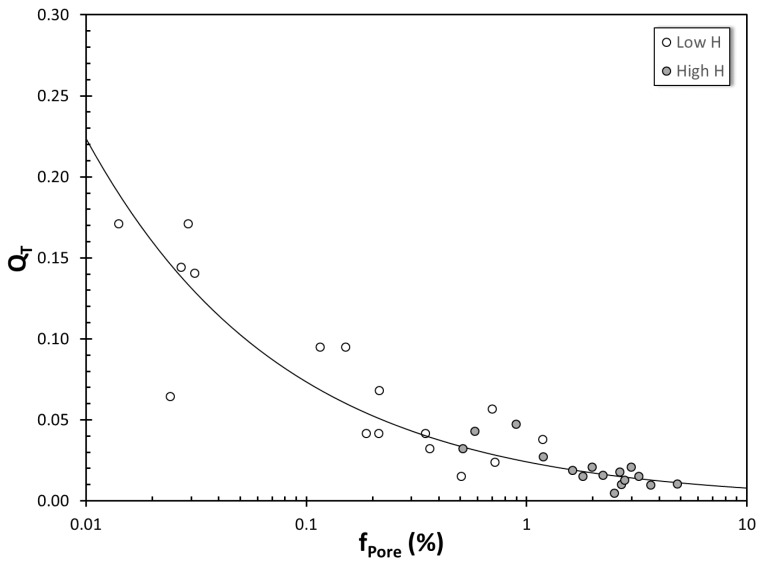
The change in Q_T_ with volumetric pore percentage in 319 datasets (data from [67]).

Let us first focus on datasets 1 and 2, which come from the same study [67]; the main difference between them is the initial hydrogen content. When Q_T_ is plotted versus volumetric percentage of pores, f_pore_, data from both high and low hydrogen groups follow the same trend, as presented in Figure 3. It is noteworthy that the extrapolation of the curve to f_Pore_ ≈ 0 gives an approximate y-intercept of 0.22. Therefore, the loss in Q_T_ of 0.78, estimated with almost no pores in the structure, is an indication of the inherent entrainment damage of the experimental (or production) system.

Recently, the author and co-workers [24] identified bifilms in A356 aluminum alloy reduced pressure test specimens that remained inactive, i.e., were not needed for the formation of pores. Building on this finding, we can expect in low-quality specimens to have a higher density of bifilms than needed to accommodate pores due to internal solidification shrinkage and/or hydrogen rejection. Consequently, a large number of bifilms remains inactive during solidification. These bifilms, because of their unbonded nature, would certainly contribute to the degradation of mechanical properties, especially elongation and fatigue life. Evidence for these bifilms that remain inactive during solidification but open up during tensile testing can be seen in in situ deformation experiments in cast Al–Si alloys [72,73]. 

**Figure 4:**
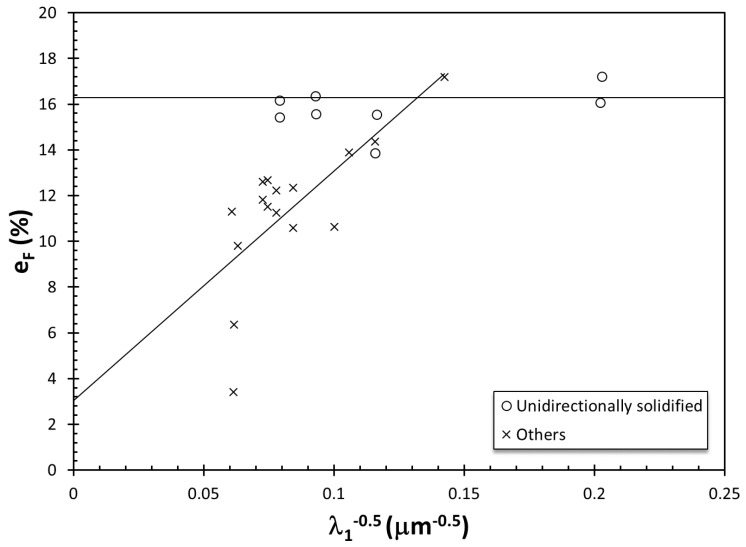
The change in elongation with the inverse square root of primary dendrite arm spacing in steel castings (data from [74]).

With decreasing damage given to the liquid metal, there would be fewer bifilms that would remain inactive during solidification, but the tensile properties would be expected to improve at lower values of λ_2_ but remain essentially the same at higher levels (longer solidification times) because of pores forming at long solidification times. Hence, the sensitivity of the metal to changing dendrite arm spacing would increase. This can be seen with maximum values in datasets 1–8 going up, whereas minima remain essentially constant, marked with a sharp increase in the slope of the correlations in Figure 2. It is also quite significant that the datasets 1–8 estimate a Q_T_ ≈ 0.04 at λ_2_ = 100 μm (1/√ λ_2_ =0.10). The effect of reduction in bifilms in the metal past this point leads to an increase in the elongation at high values of λ_2_, leading to a decrease in the slope of the correlation, as evidenced by dataset 9 in Figure 2. Further reductions in entrainment damage would lead to even more reduced slope and eventually a slope of 0, as seen in dataset 10, which are the maximum points on the yield strength–elongation, i.e., ductility, potential of cast Al–Si–Mg alloys [64]. Hence, the strength of the correlation between elongation (and therefore structural quality) and dendrite arm spacing is extrinsic, as it is determined by the bifilm content of the metal. This is consistent with the results of Polich and Flemings in [74] in cast steels, as presented in Figure 4. They found that primary dendrite arm spacing had no effect on elongation in unidirectionally solidified steel castings, in which inclusions were pushed out by columnar dendrites. In other castings, however, there was a correlation between primary dendrite arm spacing and elongation. Therefore, the λ_1_–e_F_ relationship was determined whether inclusions were allowed to be in between or in front of the growing dendrites. 

**Figure 5:**
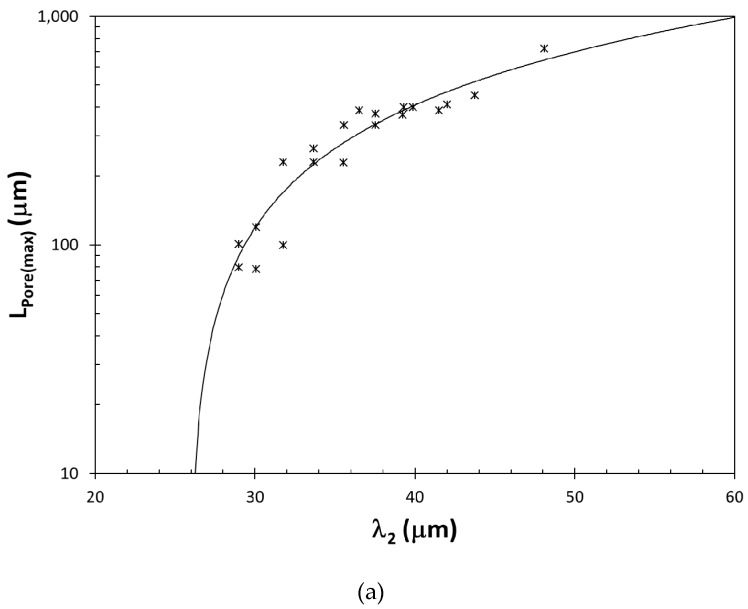

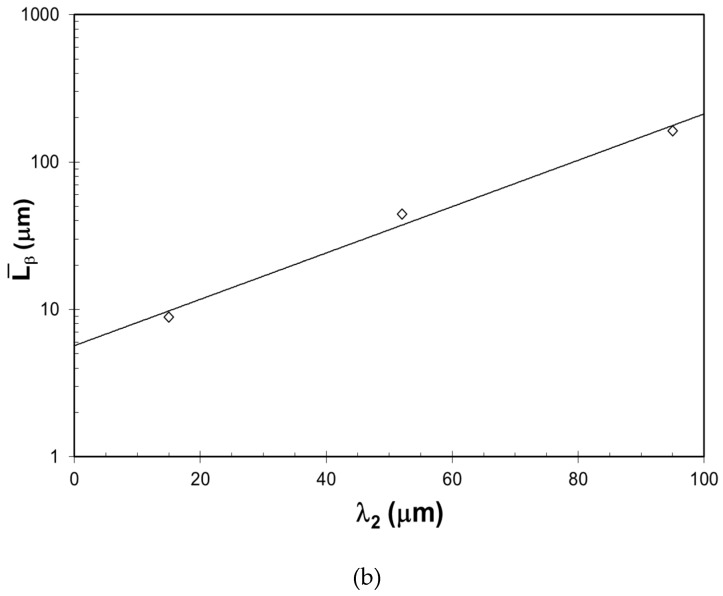
The correlation between λ_2_, and (**a**) maximum pore length (data from [75]), and (**b**) length of β–Al_5_FeSi platelets (data from [76,77]).

In general, λ_2_ can be taken as a measure of the time given to pores and intermetallics to grow. There is ample evidence for this cause-and-effect relationship in the literature. As shown in Figure 3, volumetric percentage of pores increases with λ_2_ and hydrogen content. Similarly, Zhang et al. [75] found that the length of the largest pores initiating fatigue fracture, L_Pore(max)_, increased with λ_2_ in A356 alloy castings, as presented in Figure 5a. Moreover, the length of the β–Al_5_FeSi platelets, generally thought to be weak and brittle, was also found [76,77] to increase with λ_2_, as shown in Figure 5b. Because λ_2_ changes with the 1/3 power of local solidification time [78,79,80], the degradation of elongation with increasing λ_2_ can be attributed to the growth of pores and β-platelets with time.

### 2.3. β–Al_5_FeSi Platelets

One of the curious features in the microstructure of cast aluminum alloys is the β-platelets. These platelets have the highest surface-to-volume ratio among all microstructural features, making them almost a metallurgical mystery on how they overcome the surface energy barrier for nucleation and growth. Although iron is highly soluble in liquid aluminum, it has an extremely low equilibrium solubility, i.e., 0.03 at.% (0.06 wt.%) in solid Al. As a result, the iron in solution in an aluminum alloy eventually precipitates as coarse Fe-rich intermetallic phases, such as Al_6_Fe, which precipitates in solid aluminum at approximately 300 °C [81], as compared to β–Al_5_FeSi platelets that nucleate above solidus in hypoeutectic Al–Si alloys. Under non-equilibrium conditions, up to 2 at.% (4 wt.%) Fe was found to be soluble in aluminum [82], although no Fe-bearing constituents were found with an Fe content in solution up to 10 at.% [83]. Therefore, the precipitation of β–Al_5_FeSi as platelets with enormous surface areas for their respective volumes, at temperatures above solidus, strongly implies heterogeneous nucleation [84]. In situ observations of the precipitation of β-platelets in an A356 alloy [85] showed that they nucleate parallel to secondary dendrite arms, on the oxide skin of the metal on the surface or within pores, or on existing β-platelets. All three nucleation sites can be attributed to bifilms within the liquid metal. It was shown clearly in several studies [86,87,88,89,90] that β-platelets did indeed nucleate and grow on bifilms. Liu et al. [86] verified the presence of oxygen within β-platelets in A206 alloy castings. Hence, β-platelets, just like pores, are extrinsic and act to make preexisting damage to the liquid metal visible. Because there is a bifilm inside or on one side of them, β-platelets are observed to debond and/or fracture early in plastic deformation, as observed in situ by Bjurenstedt et al. [91]. Due to the unbonded nature of the bifilm within β-platelets, Laz and Hillberry [92] even suggested that the size distribution of β-platelets be taken as the initial defect size distribution in fatigue studies of aluminum alloys.

The intrinsic properties of Fe-bearing constituents reported in the literature paint a picture that is very different from the “weak and brittle” image associated with the β-platelets. Li et al. [93,94] showed in their ab initio study that the ideal strength of FeAl is between 13.9 and 18.6 GPa, accompanied by a 14% fracture strain in tension. Although there is no study in the literature on the Al_5_FeSi phase, its ideal strength should be similar to FeAl. Seifeddine et al. [95] reported the modulus of elasticity (E) of β–Al_5_FeSi as 196 GPa. As ideal tensile strength is approximately E/10 [96], the ideal tensile strength of the β-platelets can be taken as 19.6 GPa, which is similar to the results of Li et al. Hence, β-platelets are strong and ductile; they are not weak, just extrinsically weakened by the bifilms on which they nucleate heterogeneously and grow.

### 2.4. Si Particles in Al–Si Alloys

**Figure 6:**
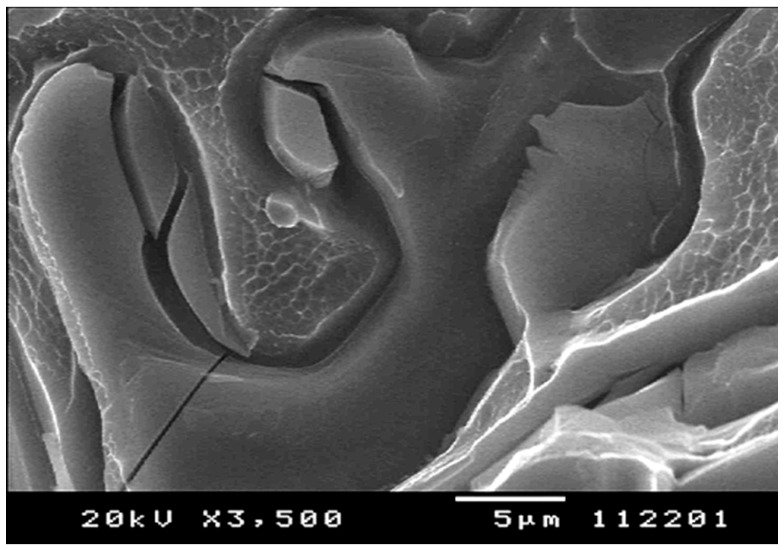
Fractured Si particles in an aerospace D357 aluminum alloy casting [102].

In cast hypoeutectic Al–Si–Mg alloys, fracture during tensile testing was observed [70] to start in the eutectic region with the appearance of microcracks. These microcracks were attributed to the fracture and/or debonding of Si eutectic particles, which lead to the formation of voids and eventually to microcracks. The damage to the Si particles, i.e., debonding and/or fracture, was found to start at 1% to 2% plastic strain [97,98] and increase linearly with plastic strain [70,99]. Usually a maximum of 10% of all Si particles were damaged in tensile or compression testing [100]. These were reported to be the largest particles [101] with the highest aspect ratios. A fractured Si particle in a tensile specimen excised from an aerospace Al–Si casting (D357) is presented in Figure 6 [102]. Note that there was extensive deformation between the particles as evidenced by very fine dimples. Moreover, Si particles were coarse, with their length exceeding 20 µm, but there was no evidence of decohesion from the aluminum matrix.

Stresses developed in the Si eutectic particles during deformation have been measured in situ in several studies. Finlayson et al. [98] reported that Si particles in Sr-modified A356 alloy castings fracture at stresses between 200 and 300 MPa at 1% strain. Finlayson et al. [98] interpreted these values as the lower bound of strength for the “weakest particles”, although they did not elaborate on the weakening mechanism. A fracture stress of 600 MPa was reported by Harris et al. [103] for a Si particle in an A319 alloy. Joseph et al. [104] investigated stress development in several Si particles in a cast Al–Si–Cu–Mg alloy with four different microstructures using the Raman technique. They found the fracture stress of the Si particles to be 0.5–1.0 GPa, with fracture strains exceeding 10%. It is noteworthy that some of the particles tracked did not fracture at all even at 30% strain. Caceres et al. [99,105] used Weibull statistics to characterize the fracture stress of Si particles and showed that particles fracture at stresses between 0.5 and 3 GPa. The cumulative probability function, P, for the Weibull distribution can be written as shown in the Equation (5) [106]:

(5) $$P=1-\exp\left[-{\left(\frac{\sigma_{\mathrm{Si}}}{\sigma_{0}}\right)}^{m}\right]$$

where σ_Si_ is the fracture stress of Si particles, σ_0_ is the scale parameter, and m is the shape parameter, alternatively referred to as the Weibull modulus. The Weibull distribution was developed based on the weakest link theory of Pierce [107], in which it is assumed that the structure is weakened by an extrinsic defect. Caceres et al. calculated σ_Si_ from tensile strain values, took *m* = 3, and obtained respectable fits to experimental data.

**Figure 7:**
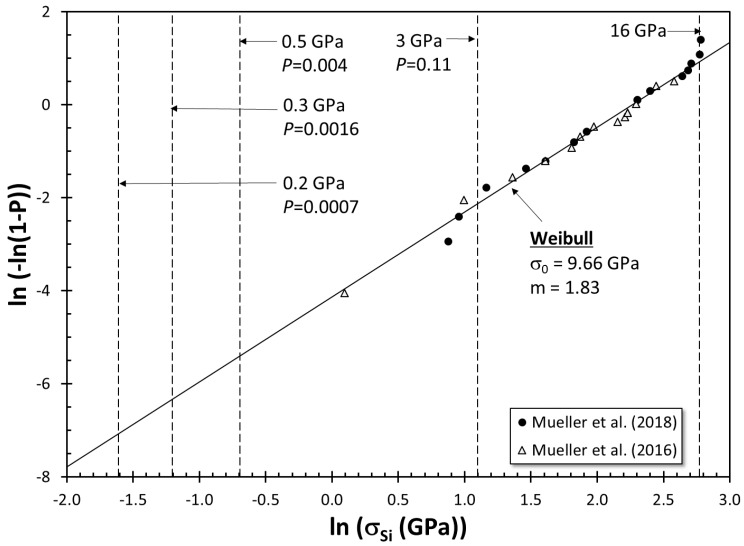
The Weibull probability plot of Si particle fracture stresses as measured by Mueller et al. (data from [108,109]).

Mueller et al. [108,109] conducted in situ micromechanical tests on the Si particles in a cast Al–12.6wt.%Si alloy. The results from the two studies by Mueller et al. are presented in a Weibull probability plot in Figure 7. Note that the stresses at which Si particles fracture are as high as 16 GPa, which is much higher than the stress levels for fracture reported in other studies. Additionally, the data for σ_Si_ are well represented by the Weibull distribution, which provides evidence that the weakest link scenario, i.e., the presence of an extrinsic weakening mechanism, applies. Mueller et al. also determined that Si particles were weakened by pinhole defects and interfaces visible on their surfaces. They also [110] found Ti- and Fe-rich intermetallics at the bottom of the pinhole defects, embedded in the Si particles, which significantly weakened the particles. In Figure 7, the limits determined by Caceres et al. are also indicated, as well as the cumulative probabilities associated with them. For stresses up to 0.5 GPa, 0.4% of Si particles are expected to crack due to extrinsic causes, and for σ_Si_ = 3 GPa, 11% of particles can be expected to fracture. The observations of Finlayson et al., σ_Si_ = 200–300 MPa, provides further evidence that Si particles are significantly weakened by other extrinsic defects, most probably by bifilms.

Several ab initio molecular dynamics studies have been conducted to estimate the ideal strength of Si. Umeno et al. [111] found the ideal strength of Si to be 16 GPa and the fracture strain to be 30% in tension. Dubois et al. [112] determined the ideal tensile strength to be 16 GPa and fracture strain as 25% in the <110> direction. The results obtained in these two studies are remarkably similar. Moreover, they are much higher than the fracture stresses reported in the experimental literature and are the same as the maximum σ_Si_ values reported by Mueller et al. for Si particles with no detectable defects. Note that the theoretical strength of 16 GPa is also indicated in Figure 7. 

Because Si is known to nucleate on bifilms [15,113] as well as other inclusions [114,115], large Si particles with high aspect ratios can be expected to have nucleated heterogeneously on bifilms during solidification before necessary undercooling is reached for homogeneous nucleation of the Si eutectic. This explains the low fracture stresses observed by Finlayson et al. When Si particles are extrinsically weakened by smaller intermetallics, they fracture at higher stress levels, as observed by Mueller et al. Therefore, Si particles are intrinsically strong enough to withstand the stresses developed during the plastic deformation of cast Al–Si alloys. 

To evaluate the debonding of Si particles, the Si–Al interface has been investigated in several studies. Xia et al. [116] determined by nanoindentations and finite element modeling that the shear strength of the Al–Si interface is 240 MPa. Ward et al. [117,118] found the interface strength in tension to be between 4 and 5 GPa via molecular dynamics simulations. Noreyan et al. [119] determined that the Si(111)/Al(111) interface had a tensile strength of 7.2 GPa but a shear strength 300 MPa, a value close to the one measured by Xia et al. In other orientations, shear strengths up to 1.2 GPa were obtained. Therefore, debonding of Si particles may be intrinsic, only when the weakest orientations occur. Because the rate of occurrence for the Si(111)/Al(111) alignment is rare, it can be concluded that most of Si debonding can be attributed to extrinsic factors.

**Figure 8:**
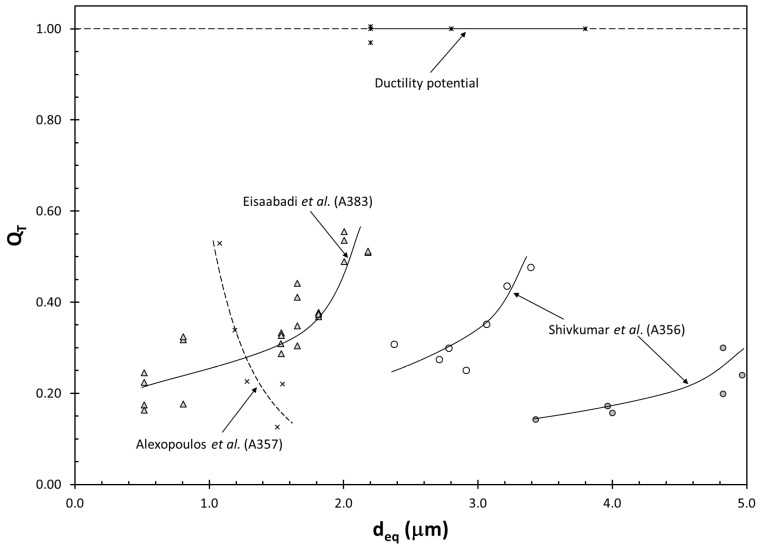
Correlations between Si particle size and ductility. The data from Shivkumar et al. are for unmodified A356 castings, with hollow points representing permanent mold castings and full points representing sand castings.

Turning our attention back to the effect of size of Si particles (d_eq_) on the ductility of cast Al alloys, results from several studies were compared. Alexopoulos et al. [120] investigated the effect of alloying additions on microstructure and tensile properties in an Al–7%Si–0.6%Mg alloy (A357), especially on the modification of the Si particles. Eisaabadi et al. [121] studied the evolution of Si particle sizes in A383 die castings with solution treatment time and its effect on tensile properties. Shivkumar et al. [122] investigated the effect of solution treatment time on the tensile properties of unmodified and modified A356 alloy permanent mold and sand castings. Data from these three studies are presented in Figure 8, along with Si particle data from maximum ductility points for Al–7%Si–Mg alloys [64]. In the studies by Eisaabadi et al. and Shivkumar et al., there is an increase in Q_T_ with increasing solution treatment time, during which Si particles coarsen [123,124]. However, note that the strength of the correlation is different in the three datasets. In A383 die castings, Si particle and Q_T_ have a strong correlation, whereas in A356 sand castings, the correlation is weak. Moreover, this trend is exactly the opposite of the Si particle size data for modification by Alexopoulos et al., in which Q_T_ increases with decreasing particle size. The reverse trends in particle size vs. quality index relationship are a clear indication that the effect of Si particle size on ductility is extrinsic, as also evidenced by the lack of any correlation from the ductility potential of cast Al–7%Si alloys.

## 3. Epilogue

It should be noted that cast Al–Si with Si contents well above the maximum solubility limit of 1.65wt.% were developed because they are more castable than those alloys with no excess Si. The reason for the higher castability is the expansion of Si upon solidification, which partially compensates for the solidification shrinkage of aluminum. With a reduction in solidification shrinkage of the alloy with increasing Si content, the magnitude of pressure drops during solidification and, therefore, the likelihood of pore formation is reduced. That is why Al–Si alloys are known as “forgiving” alloys in the foundry industry. The discussion in this paper shows that almost the entire thinking in cast aluminum alloy metallurgy is built on extrinsic features. Even the development and common use of Al–Si alloys have been due to the extrinsic features, namely entrainment defects, in castings. Therefore, our approach to aluminum castings has to be completely changed based on what the metal is intrinsically capable of producing, i.e., castings that are free from pores, hot tears, intermetallics, etc., that can perform at high levels.

## 4. Conclusions

Through analyses in this study and comparison with ideal properties, it has been determined that:

- Pores cannot nucleate, either homogeneously or heterogeneously in liquid aluminum. Therefore, nucleation is bypassed in pore formation because of the presence of bifilms in liquid aluminum, which represent the most significant, if not the only, weakening mechanism in liquid aluminum. Because pore formation is a necessary condition for the initiation of a hot tear, we can conclude that pores and hot tears are extrinsic defects and can be avoided.
- Dendrite arm spacing has no intrinsic effect on ductility. A correlation is established between ductility and dendrite arm spacing only when bifilms are present in solid aluminum. The correlation is weak at very high and very low levels of bifilm density in aluminum. At intermediate levels, the strength of the correlation first increases, then decreases with bifilm content.
- In the absence of bifilms, Fe would be retained in solution during solidification and later precipitate as Al_6_Fe in the solid aluminum matrix. Therefore, the formation of the β–Al_5_FeSi platelets takes place intrinsically due to bifilms. These platelets are intrinsically strong and ductile, but extrinsically weakened by bifilms. Therefore, subsequent fracture of β–Al_5_FeSi platelets during deformation is also extrinsic.
- Si eutectic particles are intrinsically strong and ductile, with a strength of 16 GPa as determined by molecular dynamics simulations and in situ micromechanical testing. Consequently, they should not fracture during tensile deformation unless they have precipitated on bifilms or other intermetallics. Therefore, damage to Si particles during tensile testing is extrinsic. Intrinsic debonding of Si from the aluminum matrix is possible only in very limited cases. The abundance of debonding observed in the literature suggests that extrinsic factors weaken the Al–Si interface.
- The duality in aluminum metallurgy, which is based on whether a product is cast or wrought, is a culmination of the strong, extrinsic correlations demonstrated in this paper. Even the alloys developed only for casting applications, such as the Al–Si alloys, are a result of this duality. The entire metallurgy of these alloys must be reevaluated and a new approach to the production of aluminum castings must be adopted, so that they can be produced at a lower cost and perform at much higher levels.

## Figures and Tables

**Table 1:** Details about the datasets used in Figure 2 and estimated values for coefficients of Equation (4).

Alloy	Dataset	Reference	Q_0_	Λ_0_ (μm^0.5^)	Comments
319	1	[67]	−0.0062	0.1717	High hydrogen content
	2	[67]	−0.0445	0.7928	Low hydrogen content
A380	3	[68]	−0.0490	0.4122	High pressure die cast
	4	[69]	−0.0496	0.4765	Permanent mold
A357	5	[70]	−0.2803	2.8440	No modification
	6	[70]	−0.1504	2.6931	Sr-modified
A356	7	[71]	−0.3759	4.1723	Aluminum Association dataset
	8	[70]	−0.3935	4.3961	No modification
	9	[70]	0.1626	2.4443	Sr-modified
Al–7%Si–Mg	10	[64]	1.0000	0.0000	Maximum points

## References

[1] (2003). Metallic Materials and Elements for Aerospace Vehicle Structures.

[2] Tiryakioglu M. (2019). On the Intrinsic and Extrinsic Microstructure-Property Effects in Cast Aluminum Alloys. TMS 2020 149th Annual Meeting & Exhibition Supplemental Proceedings.

[3] Staley J. (1976). Microstructure and Toughness of High-Strength Aluminum Alloys. Properties Related to Fracture Toughness.

[4] Farup I., Drezet J.-M., Rappaz M. (2001). In situ observation of hot tearing formation in succinonitrile-acetone. Acta Mater..

[5] Lu H.M., Jiang Q. (2005). Surface Tension and Its Temperature Coefficient for Liquid Metals. J. Phys. Chem. B.

[6] Yousefian P., Tiryakioglu M. (2017). Pore Formation During Solidification of Aluminum: Reconciliation of Experimental Observations, Modeling Assumptions, and Classical Nucleation Theory. Met. Mater. Trans. A.

[7] Erzi E., Tiryakioḡlu M. (2019). On the fracture pressure of liquid metals. Mater. Sci. Technol..

[8] Tiryakioglu M. (2018). On the Intrinsic Fracture Pressure of Liquid and Solid Aluminum Around Its Melting Temperature. Met. Mater. Trans. A.

[9] Campbell J. (1967). Origin of Porosity in Cast Metals. Ph.D. Thesis.

[10] Campbell J. (1968). Pore Nucleation in Solidifying Metals, Proceedings of the Conference on the Solidification of Metals, Brighton, UK, 1968.

[11] Shahani H., Fredriksson H. (1985). On the mechanism of precipitation of pores in melts. Scand. J. Metall..

[12] Tiryakioğlu M. submitted for publication.

[13] Campbell J. (2014). Cavitation in liquid and solid metals: role of bifilms. Mater. Sci. Technol..

[14] Campbell J. (2011). The Origin of Griffith Cracks. Met. Mater. Trans. A.

[15] Campbell J. (2006). Entrainment defects. Mater. Sci. Technol..

[16] Campbell J. (2006). An overview of the effects of bifilms on the structure and properties of cast alloys. Met. Mater. Trans. A.

[17] Staley J.T., Tiryakioglu M., Campbell J. (2007). The effect of increased HIP temperatures on bifilms and tensile properties of A206–T71 aluminum castings. Mater. Sci. Eng. A.

[18] Dispinar D., Campbell J. (2011). Porosity, hydrogen and bifilm content in Al alloy castings. Mater. Sci. Eng. A.

[19] Campbell J. (2014). Metallurgy without Bifilms: No More Fractures, Proceedings of the Shape Casting: 5th International Symposium 2014.

[20] El-Sayed M., Griffiths W.D. (2014). Hydrogen, bifilms and mechanical properties of Al castings. Int. J. Cast Met. Res..

[21] El-Sayed M.A., Hassanin H., Essa K., El-Sayed M. (2016). Bifilm defects and porosity in Al cast alloys. Int. J. Adv. Manuf. Technol..

[22] Tunçay T., Tekeli S., Özyürek D., Dişpinar D. (2016). Microstructure–bifilm interaction and its relation with mechanical properties in A356. Int. J. Cast Met. Res..

[23] Uludağ M., Çetin R., Dispinar D., Tiryakioglu M. (2017). Characterization of the Effect of Melt Treatments on Melt Quality in Al–7wt %Si–Mg Alloys. Metals.

[24] Tiryakioglu M., Yousefian P., Eason P.D. (2018). Quantification of Entrainment Damage in A356 Aluminum Alloy Castings. Met. Mater. Trans. A.

[25] Dispinar D., Campbell J. (2004). Critical assessment of reduced pressure test. Part 1: Porosity phenomena. Int. J. Cast Met. Res..

[26] Dispinar D., Campbell J. (2004). Critical assessment of reduced pressure test. Part 2: Quantification. Int. J. Cast Met. Res..

[27] Uludağ M., Çetin R., Dişpinar D., Tiryakioglu M., Tiryakioǧlu M. (2018). The effects of degassing, grain refinement & Sr-addition on melt quality-hot tear sensitivity relationships in cast A380 aluminum alloy. Eng. Fail. Anal..

[28] Campbell J. (2015). Complete Casting Handbook: Metal. Casting Processes, Metallurgy, Techniques and Design.

[29] Campbell J. (2012). “Stop Pouring, Start Casting”. Int. J. Met..

[30] Tiryakioḡlu M. (2020). The Effect of Hydrogen on Pore Formation in Aluminum Alloy Castings: Myth Versus Reality. Metals.

[31] Zhang J., Singer R.F. (2004). Effect of grain-boundary characteristics on castability of nickel-base superalloys. Met. Mater. Trans. A.

[32] Lin S., Aliravci C., Pekguleryuz M. (2007). Hot-Tear Susceptibility of Aluminum Wrought Alloys and the Effect of Grain Refining. Met. Mater. Trans. A.

[33] Easton M., Wang H., Grandfield J., St John D., Sweet E. (2004). In An analysis of the effect of grain refinement on the hot tearing of aluminium alloys. Mater. Forum.

[34] Schumacher P., Greer A.L. (1994). Heterogeneously nucleated α–Al in amorphous aluminium alloys. Mater. Sci. Eng. A.

[35] Mohanty P., Gruzleski J. (1995). Mechanism of grain refinement in aluminium. Acta Met. et Mater..

[36] Schumacher P., Greer A.L., Worth J., Evans P.V., Kearns M.A., Fisher P., Green A.H. (1998). New studies of nucleation mechanisms in aluminium alloys: Implications for grain refinement practice. Mater. Sci. Technol..

[37] Easton M., StJohn D. (1999). Grain refinement of aluminum alloys: Part II. Confirmation of, and a mechanism for, the solute paradigm. Met. Mater. Trans. A.

[38] Easton M., StJohn D. (1999). Grain refinement of aluminum alloys: Part I. the nucleant and solute paradigms—a review of the literature. Met. Mater. Trans. A.

[39] Greer A.L., Bunn A., Tronche A., Evans P., Bristow D. (2000). Modelling of inoculation of metallic melts: application to grain refinement of aluminium by Al–Ti–B. Acta Mater..

[40] Easton M., StJohn D. (2005). An analysis of the relationship between grain size, solute content, and the potency and number density of nucleant particles. Met. Mater. Trans. A.

[41] Dahle A., StJohn D. (1998). Rheological behaviour of the mushy zone and its effect on the formation of casting defects during solidification. Acta Mater..

[42] Bishop H., Myskowski E., Pellini W. (1951). Contribution of riser and casting end effects to the soundness of steel bars. AFS Trans..

[43] Bishop H., Pellini W. (1950). The contribution of risers and chill-edge effects to soundness of cast steel plates. AFS Trans..

[44] Myskowski E., Bishop H., Pellini W. (1953). Feeding range of joined sections. AFS Trans..

[45] Pellini W.S. (1953). Factors which determine riser adequacy and feeding range. AFS Trans..

[46] Tiryakioglu M. (2019). On intrinsic and extrinsic feeding distance calculations for aluminium alloys. Mater. Sci. Technol..

[47] Erzi E., Tiryakioğlu M. (2019). Feeding distance of tin bronze castings: intrinsic and extrinsic estimates. Mater. Sci. Technol..

[48] Mikhailovskij I., Mazilova T.I., Voyevodin V.N., Mazilov A. (2011). Inherent strength of grain boundaries in tungsten. Phys. Rev. B.

[49] Kotrechko S., Ovsjannikov O., Mazilova T., Mikhailovskij I., Sadanov E., Stetsenko N. (2017). Inherent hydrostatic tensile strength of tungsten nanocrystals. Philos. Mag..

[50] Fridman V.Y. (1971). Strength and plasticity of submicron tungsten fibers. Sov. Phys. Solid State.

[51] Rasool H.I., Ophus C., Klug W.S., Zettl A., Gimzewski J.K. (2013). Measurement of the intrinsic strength of crystalline and polycrystalline graphene. Nat. Commun..

[52] Lee G.-H., Cooper R., An S.J., Lee S., Van Der Zande A.M., Petrone N., Hammerberg A.G., Lee C., Crawford B., Oliver W. (2013). High-Strength Chemical-Vapor-Deposited Graphene and Grain Boundaries. Science.

[53] Lu G.-H., Deng S., Wang T., Kohyama M., Yamamoto R. (2004). Theoretical tensile strength of an Al grain boundary. Phys. Rev. B.

[54] Zhang S., Kontsevoi O.Y., Freeman A.J., Olson G.B. (2010). Sodium-induced embrittlement of an aluminum grain boundary. Phys. Rev. B.

[55] Lu G.-H., Zhang Y., Deng S., Wang T., Kohyama M., Yamamoto R., Liu F., Horikawa K., Kanno M. (2006). Origin of intergranular embrittlement of Al alloys induced by Na and Ca segregation: Grain boundary weakening. Phys. Rev. B.

[56] Lu G.-H., Suzuki A., Ito A., Kohyama M., Yamamoto R. (2001). Ab initio pseudopotential studies on an Al Σ = 9 grain boundary: Effects of Na and Ca impurities. Philos. Mag. Lett..

[57] Flemings M.C. (1974). Solidification processing. Met. Mater. Trans. A.

[58] Reyes R.V., Kakitani R., Costa T., Spinelli J.E., Cheung N., Garcia A. (2016). Cooling thermal parameters, microstructural spacing and mechanical properties in a directionally solidified hypereutectic Al–Si alloy. Philos. Mag. Lett..

[59] Reyes R.V., Bello T.S., Kakitani R., Costa T., Garcia A., Cheung N., Spinelli J.E. (2017). Tensile properties and related microstructural aspects of hypereutectic Al–Si alloys directionally solidified under different melt superheats and transient heat flow conditions. Mater. Sci. Eng. A.

[60] Duarte R.N., Faria J.D., Brito C., Veríssimo N.C., Cheung N., Garcia A. (2016). Length scale of the dendritic microstructure affecting tensile properties of Al–(Ag)–(Cu) alloys. Int. J. Mod. Phys. B.

[61] Canté M.V., Spinelli J.E., Cheung N., Garcia A. (2010). The correlation between dendritic microstructure and mechanical properties of directionally solidified hypoeutectic Al–Ni alloys. Met. Mater. Int..

[62] Tiryakioclu M., Campbell J., Tiryakioglu M. (2009). Ductility, structural quality, and fracture toughness of Al–Cu–Mg–Ag (A201) alloy castings. Mater. Sci. Technol..

[63] Tiryakioğlu M., Campbell J., Alexopoulos N.D. (2009). Quality Indices for Aluminum Alloy Castings: A Critical Review. Met. Mater. Trans. A.

[64] Tiryakioğlu M., Campbell J., Alexopoulos N.D. (2009). On the Ductility of Cast Al–7 Pct Si–Mg Alloys. Met. Mater. Trans. A.

[65] Tiryakioğlu M., Campbell J., Alexopoulos N.D. (2009). On the ductility potential of cast Al–Cu–Mg (206) alloys. Mater. Sci. Eng. A.

[66] Tiryakioğlu M., Campbell J. (2014). Quality Index for Aluminum Alloy Castings. Int. J. Met..

[67] Samuel A.M., Samuel F.H. (1995). Effect of melt treatment, solidification conditions and porosity level on the tensile properties of 319.2 endchill aluminium castings. J. Mater. Sci..

[68] Grosselle F., Timelli G., Bonollo F., Molina R. (2009). Correlation between microstructure and mechanical properties of Al–Si diecast engine blocks. Metall. Sci. Technol..

[69] Grosselle F., Timelli G., Bonollo F., Tiziani A., Della Corte E. (2009). Correlation between microstructure and mechanical properties of Al–Si cast alloys. Metall. Ital..

[70] Wang Q. (2003). Microstructural effects on the tensile and fracture behavior of aluminum casting alloys a356/357. Metall. Mater. Trans. A.

[71] Miguelucci E. (1985). The aluminum association cast alloy test program: Interim report. AFS Trans..

[72] Doglione R. (2012). In Situ Investigations on the Ductility of an Al–Si–Mg Casting Alloy. JOM.

[73] Ghassemali E., Riestra M., Bogdanoff T., Kumar B.S., Seifeddine S. (2017). Hall-Petch equation in a hypoeutectic Al–Si cast alloy: grain size vs. secondary dendrite arm spacing. Procedia Eng..

[74] Polich R., Flemings M. (1965). Mechanical properties of unidirectional steel castings. AFS Trans..

[75] Zhang B., Chen W., Poirier D.R. (2000). Effect of solidification cooling rate on the fatigue life of A356.2–T6 cast aluminium alloy. Fatigue Fract. Eng. Mater. Struct..

[76] Vorren O., Evensen J., Pedersen T. (1984). Microstructure and mechanical properties of AlSi (Mg) casting alloys. AFS Trans..

[77] Samuel A.M., Samuel F.H., Doty H.W. (1996). Observations on the formation of β–Al_5_FeSi phase in 319 type Al–Si alloys. J. Mater. Sci..

[78] Kirkwood D. (1985). A simple model for dendrite arm coarsening during solidification. Mater. Sci. Eng..

[79] Feurer U., Wunderlin R. (1977). Einfluss der Zusammensetzung und der Erstarrungsbedingungen auf die Dendritenmorphologie binärer Al-Legierungen, Fachbericht der Deutschen Gesellschaft für Metallkunde.

[80] Tiryakioğlu M. (2019). A Simple Model to Estimate Solidification Time–Dendrite Arm Spacing Relationships in Cast Aluminum Alloys with Two Major Alloying Additions: Application to the Al–Si–Cu System. Met. Mater. Trans. A.

[81] Saller B.D., Hu T., Ma K., Kurmanaeva L., Lavernia E., Schoenung J. (2015). A comparative analysis of solubility, segregation, and phase formation in atomized and cryomilled Al–Fe alloy powders. J. Mater. Sci..

[82] Saller B.D., Sha G., Yang L.M., Liu F., Ringer S., Schoenung J.M. (2017). Iron in solution with aluminum matrix after non-equilibrium processing: an atom probe tomography study. Philos. Mag. Lett..

[83] Nayak S., Wollgarten M., Banhart J., Pabi S.K., Murty B. (2010). Nanocomposites and an extremely hard nanocrystalline intermetallic of Al–Fe alloys prepared by mechanical alloying. Mater. Sci. Eng. A.

[84] Shuey R.T. (2008). Personal Communication.

[85] Puncreobutr C., Phillion A., Fife J., Rockett P., Horsfield A., Lee P.D. (2014). In situ quantification of the nucleation and growth of Fe-rich intermetallics during Al alloy solidification. Acta Mater..

[86] Liu K., Cao X., Chen X.-G. (2010). Solidification of Iron-Rich Intermetallic Phases in Al–4.5Cu–0.3Fe Cast Alloy. Met. Mater. Trans. A.

[87] Cao X., Campbell J. (2004). The solidification characteristics of Fe-rich intermetallics in Al–11.5Si–0.4Mg cast alloys. Met. Mater. Trans. A.

[88] Cao X., Campbell J. (2003). The nucleation of Fe-Rich phases on oxide films in Al–11.5Si–0.4Mg cast alloys. Met. Mater. Trans. A.

[89] Cao X., Campbell J. (2006). Morphology of β–Al5FeSi Phase in Al–Si Cast Alloys. Mater. Trans..

[90] Miller D.N., Lu L., Dahle A.K. (2006). The role of oxides in the formation of primary iron intermetallics in an Al–11.6Si–0.37Mg alloy. Met. Mater. Trans. A.

[91] Bjurenstedt A., Ghassemali E., Seifeddine S., Dahle A.K. (2019). The effect of Fe-rich intermetallics on crack initiation in cast Aluminium: An in-situ tensile study. Mater. Sci. Eng. A.

[92] Laz P.J., Hillberry B. (1998). Fatigue life prediction from inclusion initiated cracks. Int. J. Fatigue.

[93] Li T., Morris J.W., Chrzan D.C. (2006). Ab initio study of the ideal shear strength and elastic deformation behaviors of B2 FeAl and NiAl. Phys. Rev. B.

[94] Li T., Morris J.W., Chrzan D.C. (2004). Ideal tensile strength of B2 transition-metal aluminides. Phys. Rev. B.

[95] Seifeddine S., Johansson S., Svensson I.L. (2008). The influence of cooling rate and manganese content on the β–Al5FeSi phase formation and mechanical properties of Al–Si-based alloys. Mater. Sci. Eng. A.

[96] Zhu T., Li J., Ogata S., Yip S. (2009). Mechanics of Ultra-Strength Materials. MRS Bull..

[97] Gangulee A., Gurland J. (1967). On the fracture of silicon particles in aluminum- silicon alloys. Trans. Metall. Soc. AIME.

[98] Finlayson T., Griffiths J., Viano D., Fitzpatrick M., Oliver E., Wang Q. (2007). In Stresses in the eutectic silicon particles of strontium-modified a356 castings loaded in tension. Shape Casting: 2nd International Symposium, Proceedings of the Shape Casting: 2nd International Symposium, Warrendale, PA, USA, 2007.

[99] Cáceres C., Griffiths J. (1996). Damage by the cracking of silicon particles in an Al–7Si–0.4Mg casting alloy. Acta Mater..

[100] Yeh J.-W., Liu W.-P. (1996). The cracking mechanism of silicon particles in an A357 aluminum alloy. Met. Mater. Trans. A.

[101] Poole W., Charras N. (2005). An experimental study on the effect of damage on the stress–strain behaviour for Al–Si model composites. Mater. Sci. Eng. A.

[102] Tiryakioğlu M. (2002). Tensile Deformation, Fracture and Hardness Characteristics of cast Al–7wt.%Si–Mg alloys. Ph.D. Thesis.

[103] Harris S.J., O’Neill A., Boileau J., Donlon W., Su X., Majumdar B. (2007). Application of the Raman technique to measure stress states in individual Si particles in a cast Al–Si alloy. Acta Mater..

[104] Joseph S., Kumar S., Bhadram V.S., Narayana C. (2015). Stress states in individual Si particles of a cast Al–Si alloy: Micro-Raman analysis and microstructure based modeling. J. Alloy. Compd..

[105] Wang Q.G., Caceres C.H., Griffiths J.R. (2003). Damage by eutectic particle cracking in aluminum casting alloys a356/357. Metall. Mater. Trans. A.

[106] Weibull W. (1951). A statistical distribution function of wide applicability. J. Appl. Mech..

[107] Pierce F. (1926). Tensile tests for cotton yarns: V. The “weakest link” theorems on the strength of long and of composite specimens. J. Text. Inst..

[108] Mueller M., Žagar G., Mortensen A. (2018). In-situ strength of individual silicon particles within an aluminium casting alloy. Acta Mater..

[109] Mueller M., Fornabaio M., Žagar G., Mortensen A. (2016). Microscopic strength of silicon particles in an aluminium–silicon alloy. Acta Mater..

[110] Mueller M., Fornabaio M., Mortensen A. (2016). Silicon particle pinhole defects in aluminium–silicon alloys. J. Mater. Sci..

[111] Umeno Y., Kushima A., Kitamura T., Gumbsch P., Li J. (2005). Ab initiostudy of the surface properties and ideal strength of (100) silicon thin films. Phys. Rev. B.

[112] Dubois S.M.-M., Rignanese G.-M., Pardoen T., Charlier J.-C. (2006). Ideal strength of silicon: An ab initio study. Phys. Rev. B.

[113] Campbell J., Tiryakioğlu M. (2006). Modelling Microstructure and Properties: The Contributions of Grain Size, DAS and Bifilms. Mater. Sci. Forum.

[114] Shankar S., Riddle Y.W., Makhlouf M.M. (2004). Nucleation mechanism of the eutectic phases in aluminum–silicon hypoeutectic alloys. Acta Mater..

[115] Shankar S., Riddle Y.W., Makhlouf M.M. (2004). Eutectic solidification of aluminum-silicon alloys. Met. Mater. Trans. A.

[116] Xia S., Qi Y., Perry T., Kim K.-S. (2009). Strength characterization of Al/Si interfaces: A hybrid method of nanoindentation and finite element analysis. Acta Mater..

[117] Ward D., Curtin W., Qi Y. (2006). Aluminum–silicon interfaces and nanocomposites: A molecular dynamics study. Compos. Sci. Technol..

[118] Ward N.K., Curtin W., Qi Y. (2006). Mechanical behavior of aluminum–silicon nanocomposites: A molecular dynamics study. Acta Mater..

[119] Noreyan A., Qi Y., Stoilov V. (2008). Critical shear stresses at aluminum–silicon interfaces. Acta Mater..

[120] Alexopoulos N.D., Tiryakioğlu M., Vasilakos A.N., Kourkoulis S.K. (2014). The effect of Cu, Ag, Sm and Sr additions on the statistical distributions of Si particles and tensile properties in A357–T6 alloy castings. Mater. Sci. Eng. A.

[121] Yeom G.Y., Tiryakioğlu M., Netto N., Beygi R., Mehrizi M.Z., Kim S.K. (2018). The effect of solution treatment time on the microstructure and ductility of naturally-aged A383 alloy die castings. Mater. Sci. Eng. A.

[122] Shivkumar S., Ricci S., Keller C., Apelian D. (1990). Effect of solution treatment parameters on tensile properties of cast aluminum alloys. J. Heat Treat..

[123] Tiryakioğlu M. (2008). Si particle size and aspect ratio distributions in an Al–7%Si–0.6%Mg alloy during solution treatment. Mater. Sci. Eng. A.

[124] Rhines F.N., Aballe M. (1986). Growth of silicon particles in an aluminum matrix. Met. Mater. Trans. A.

